# Remote monitoring contributes to preventing overwork-related events in health workers at the COVID-19 frontlines

**DOI:** 10.1093/pcmedi/pbaa014

**Published:** 2020-05-11

**Authors:** Faming Zhang, Huiquan Wang, Ruijuan Chen, Wenzhi Hu, Yuexia Zhong, Xin Wang

**Affiliations:** 1 Medical Center for Digestive Diseases, Second Affiliated Hospital of Nanjing Medical University, Nanjing, China; 2 Biomedical Engineering Department, School of Life Sciences, Tiangong University, Tianjin, China; 3 Department of Cardiology, Second Affiliated Hospital of Nanjing Medical University, Nanjing, China; 4 Wuhan Huoshenshan Hospital, Wuhan, China; 5 Tangdu Hospital, Fourth Military Medical University, Xi'an, China

**Keywords:** coronavirus, COVID-19, overwork, sudden death, wearable device, 5 G

## Abstract

Fighting at the frontlines against the coronavirus disease 2019 (COVID-19) pandemic, the health workers are at high risk of virus infection and overwork-related sudden death and disorders including cardiovascular diseases and stress. When we noticed the increase of overwork-related sudden deaths in physicians and nurses in the first two weeks after lockdown of Wuhan, we organized the ‘Touching Your Heart’ program by remote monitoring, aiming to protect health workers from overwork-related disorders through integrated volunteer work by physicians and medical engineering researchers from Wuhan Huoshenshan Hospital, Nanjing Medical University and Tiangong University. By remotely monitoring the health condition of the medical aid team working at Wuhan Huoshenshan Hospital, the program successfully helped in avoiding severe overwork-related events in them. The result of our program reminded the frontline health workers around the world to take precaution against overworked-related severe events, and showed that precision monitoring is effective in improving work efficiency and maintaining sustainable work force during the emergency situations like a pandemic.

**The high risk of overwork-related events during COVID-19 pandemic**

The coronavirus disease 2019 (COVID-19), which started in December 2019 and quickly spread around the world, had more than 2,200,000 confirmed cases up to April 18th, 2020. The physicians, nurses and other health workers are at the frontlines of the pandemic, working in great tension and pressure, committing their lives to controlling and preventing spread of the disease. In China for example, since the lockdown of Wuhan on January 23rd, 2020, most hospitals and health workers have been in an emergency work mode. The Chinese government organized over 42,000

physicians, nurses and medical technicians outside Hubei to work with 500,000 local health professionals in the province [1]. Among them, 1,415 persons including physicians, nurses and other staffs worked at the Wuhan Huoshenshan Hospital with 1000 beds, which was built within 10 days after the lockdown of Wuhan city, mainly for treating severe and critical COVID-19 cases.

Due to lack of health workers and personal protective equipment (PPE), most of the doctors and nurses have to work for a long time while wearing protective clothes and appliances. Hypoxia, sweat loss, insufficient energy and inconvenience to go to toilet are the main problems under the work status with sealed protective appliances in the ward. They are therefore at high risk of overwork-related sudden death and disorders including cardiovascular diseases.

Besides the physical conditions resulting from heavy work burden, the fear and panic caused by the outbreak itself and the control measures is another problem facing the medical staff.[2,3] The psychological stress adds to the physical fatigue and together they may result in serious health events in health workers. While the pandemic in China has been successfully controlled, we are sorry to lose 18 health workers to overwork related events, which took place at 24.89 ± 3.67 days (range 4 - 56 days) after the lockdown, at an average age of 47.17 ± 2.54 (range 28-69) years old.

**The "Touching Your Heart" program for frontline physicians and nurses**

When we noticed the increase of overworked-related sudden deaths in the first two weeks after lockdown of Wuhan City, we attempted to organize researchers and physicians out of hospital managing COVID-19 patients to set new technological method for the early diagnosis of severe heart events by remote monitoring.

Therefore, we organized the “Touching Your Heart” program, which by integrating physicians and medical engineering researchers from universities as a volunteer team, aimed to protect health workers from overwork-related disorders. The cooperation involved Xin Wang’s medical aid team at Huoshenshan Hospital in Wuhan, Faming Zhang’s physicians team at the Second Affiliated Hospital of Nanjing Medical University in Nanjing, and Huiquan Wang’s engineering team at Tiangong University in Tianjin. This monitoring program was approved by the ethical committee of Wuhan Huoshenshan Hospital. All participants were voluntary, informed and agreeing to cooperate in the whole process. A set of wearable devices and online fatigue surveys were developed to remotely monitor the physical condition of the health workers by 5G network, which refers to the fifth generation of wireless transmission technology (Fig. 1). The 5G networks has shown great advantages in hospital intelligence services, including allowing automatic patient monitoring via wearable devices carried by patients.[4] The wearable monitoring devices in our program would not be hampered by the protecting clothes, sweat, or work performance of the physicians and nurses.

## Remote monitoring warning against overwork in health workers

The remote monitoring integrated multi-source information including the electrocardiogram, work load, work time, and fatigue level, to warn against the overwork-related cardiac events. The design was that the monitoring system could identify those physicians or nurses who were overworked and should be exchanged out for rest or further treatment. All together we collected 153 times of monitoring for physicians and nurses at work and in rest. Myocardial ischemia complicated arrhythmia was observed in a physician in his 50s. Subsequent coronary computer tomography arteriography showed more than 80% coronary artery stenosis. One nurse was monitored to have frequent ventricular premature beats. They were required to quit work for treatment. Arrhythmia was found for 40 times in the aid team.

We observed that the heart rate of physicians and nurses peaked at 8:00-14:00 (106 ± 17.45 bpm; Fig. 2). The work schedule and service force were updated by nursing division based on the present integrated analysis, which might contribute to improving the work efficiency and reducing the overworked-related serious events for nurses. By doing so, the Touching Your Heart program contributed in preventing overwork-related severe events in the medical aid team.

## Conclusions

With the development of pandemic, the soaring confirmed and suspected cases present enormous workload for the global health workers, policemen and other staff on the frontlines, who will have to face such challenges for at least a few more months. To fight the pandemic, we need to have sustainable work force in these teams. Our “Touching Your Heart” program was a pilot of taking care of their health through remote monitoring. Such precision monitoring show promise in improving work efficiency and maintaining sustainable work force during times of emergency like a pandemic.

**Competing interests**: All authors declare no competing interests.

**Acknowledgements:** We appreciate the professional assistance from Pei Li and Nan Zhang from Wuhan Huoshenshan Hospital, Feng Li, Min Dai, Xiao Ding, Weihong Wang and Yixin Yao from the Second Affiliated Hospital of Nanjing Medical University, and Meng Hu, Guanhua Deng, Xiaofei Diao, Shumian Xiao, Xu Han, Meng Guo and Xin Lian from Tiangong University. This program was partially supported by Tianjin Science and Technology Plan Project (No.18ZXRHSY00200) and Jiangsu Provincial Medical Innovation Team (Zhang F).

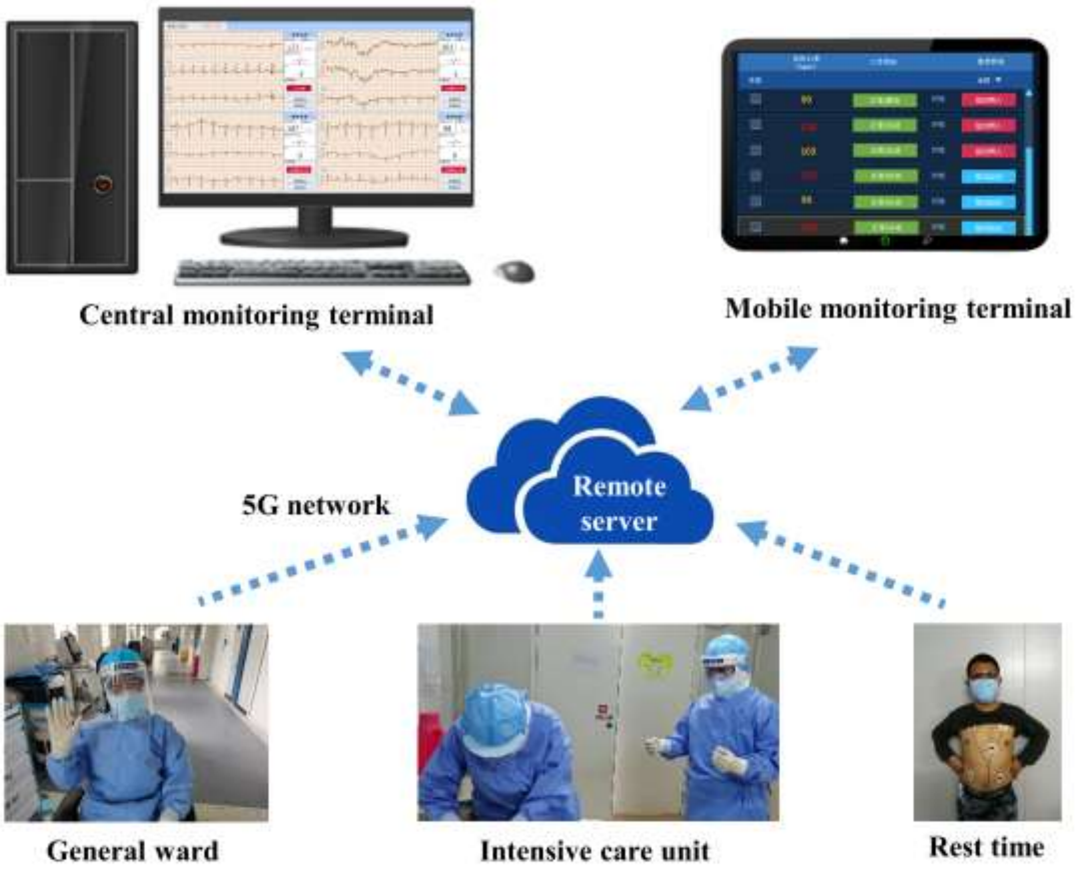

Figure 1: The schematic drawing illustrating the “Touching Your Heart” program.

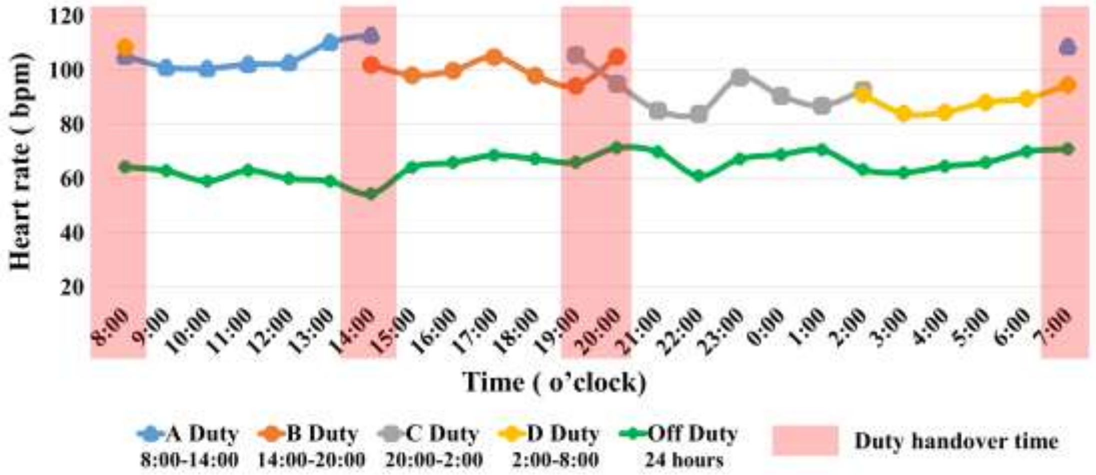

Figure 2: The heart rates changes of the nurses and physicians caring COVID-19 patients.
